# Lingual thyroid with severe hypothyroidism: A case report

**DOI:** 10.1097/MD.0000000000027612

**Published:** 2021-10-29

**Authors:** Hsuan Huang, Yi-Hsin Lin

**Affiliations:** a Division of Pediatric Surgery, Department of Surgery, Mackay Memorial Hospital, Taipei, Taiwan (R.O.C.); b Division of Endocrinology and Metabolism, Department of Internal Medicine, Taiwan Adventist Hospital, Taipei, Taiwan (R.O.C.).

**Keywords:** ectopic thyroid tissue, hypothyroidism, lingual thyroid

## Abstract

**Introduction:**

Ectopic thyroid tissue presenting at the base of the tongue, called lingual thyroid, is a clinical rarity. Clinical presentation varies depending upon either the severity of regional symptoms associated with the enlargement of gland size, or the features related to thyroid dysfunction.

**Patient concerns:**

We reported a case of a 29-year-old female who presented with symptoms of easy fatigue and depression for 3 months.

**Diagnosis:**

After a series of diagnostic workup, the lingual thyroid with severe hypothyroidism was diagnosed.

**Intervention and outcome:**

She received conservative treatment with thyroid hormone replacement and the symptoms improved significantly.

**Lessons:**

Lingual thyroid is a rare entity that needs careful diagnostic workup including clinical examination, biochemical tests, imaging methods such as ultrasonography, scintigraphy, computed tomography, magnetic resonance imaging, and fine-needle aspiration cytology to plan the management. Lingual thyroid with hypothyroidism and no neck regional symptoms can be conservatively treated and requires regular follow-up for the prevention of potential risk of malignant transformation.

## 1 Introduction

The normal thyroid gland is located at the front of the neck, below the thyroid cartilage. In humans, thyroid dysgenesis means the disturbed organogenesis of the thyroid gland, that leads to a variety of conditions, such as agenesis, hypoplasia, and ectopy. The most common disorder of thyroid dysgenesis is ectopic thyroid tissue (ETT). ETT is the presence of thyroid tissue outside its normal anatomical position, due to its embryonic failure to descend from the base of the tongue to the mediastinum, along the path of the obliterated thyroglossal duct. ETT is relatively rare with an overall prevalence of 1 in 100,000 to 300,000 persons.^[1]^ While ETT presents at the base of the tongue, it is called lingual thyroid which accounts for 90% of all ETT. Lingual thyroid occurs more commonly in females (70%–80%).^[2–4]^ Most cases of lingual thyroid are asymptomatic unless a progressive enlargement of gland size resulting in dysphagia, foreign body sensation or pain in the throat, dysphonia, bleeding, and even dyspnea or features related to thyroid dysfunction.^[3,5]^ The diagnosis of lingual thyroid is usually through clinical examination, biochemical tests, imaging methods including ultrasonography, thyroid scintigraphy, computed tomography (CT) scan, magnetic resonance imaging (MRI), and fine-needle aspiration cytology (FNAC) plays as a key role in diagnosing confirmation.^[1,6,7]^ We reported a case of a 29-year-old female who presented with symptoms of easy fatigue and depression for 3 months. After a series of diagnostic workup, the lingual thyroid with severe hypothyroidism was diagnosed. The article aims to report a rare clinical entity of lingual thyroid, its presentation, diagnosis, management, and review of the literature.

## 2 Consent for publication

Written informed consent was obtained from the patient for publication of this case report and accompanying images. This case report was conducted under the Declaration of Helsinki. It was approved by the Institutional Review Board of Taiwan Adventist Hospital.

## 3 Case report

**Figure 1:**
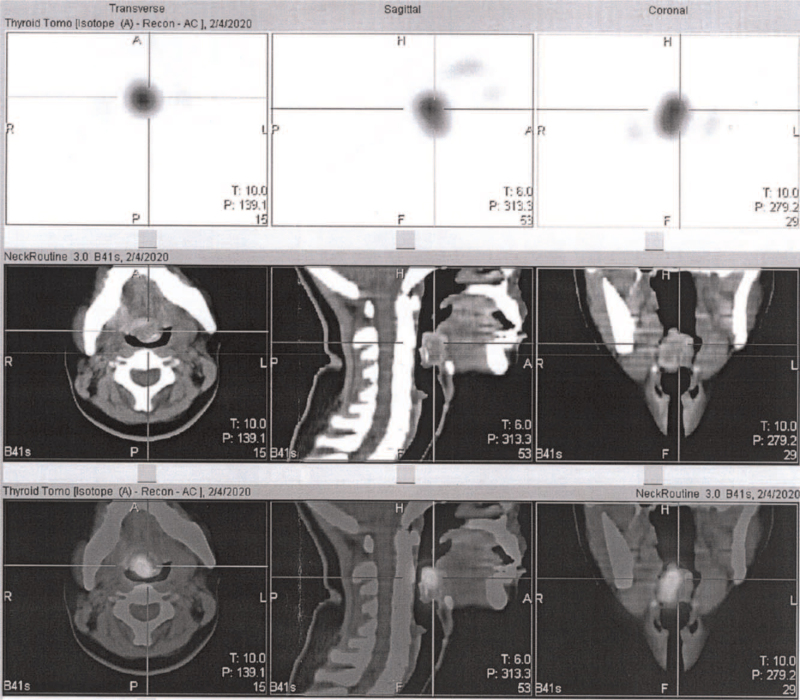
Thyroid scintigraphy with technetium (^99m^Tc) showed uptake of the radiotracer at the base of the tongue. Lingual thyroid was detected. No uptake was seen in the normal thyroid gland location.

**Figure 2:**
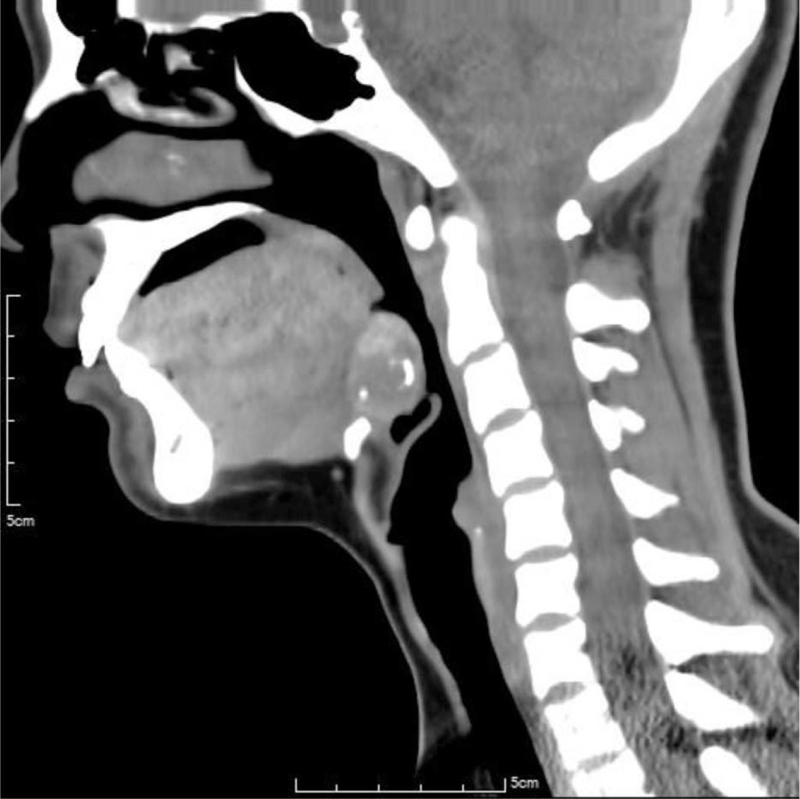
Non-contrast computed tomography scan of the neck showed a well-defined, mixed hyperdense and hypodense nodule, measuring 26 × 24 × 19 mm in the midline at the tongue base with focal ring calcification, suggestive of lingual thyroid. No thyroid gland was seen in the orthotopic location.

A 29-year-old female presented to the outpatient department with complaints of easy fatigue and depression for 3 months. She did not have any congenital or systemic diseases in the past. The laboratory tests revealed normal data except for hypothyroidism (TSH 18.8 uIU/mL [reference value 0.38–5.33 uIU/mL], free T4 0.72 ng/dL [reference value 0.70–1.80 ng/dL], and T3 123 ng/dL [reference value 78–153 ng/dL]). Her thyroid autoimmune antibodies (thyroglobulin antibody and anti-TPO antibody) were negative and serum thyroglobulin level was within the normal range (21.89 ng/mL [reference value <50 ng/mL]). Ultrasonography for the thyroid and neck revealed the absence of a thyroid gland in its orthotopic location. Thyroid scintigraphy with technetium (^99m^Tc) showed uptake of the radiotracer at the base of the tongue. Lingual thyroid was detected. No uptake was seen in the normal thyroid gland location (Fig. 1). Further CT scan of the neck showed a well-defined, mixed hyperdense and hypodense nodule, measuring 26 × 24 × 19 mm at the tongue base with focal ring calcification, suggestive of lingual thyroid. No thyroid gland was seen in the orthotopic location (Fig. 2). The diagnosis of this patient is lingual thyroid with severe hypothyroidism. FNAC for the lingual thyroid was suggested to rule out malignancy potential, but the patient hesitated and received conservative treatment with levothyroxine 100 μg supplement once daily. Her thyroid function returned to euthyroidism in 2 months, and she had significant improvement in symptoms. Now her condition is stable on regular follow-up for already 3 years.

## 4 Discussion

The thyroid is an endocrine gland in the human body. It is normally located in the lower part of the anterior neck region inferior to the thyroid cartilage. The thyroid contains 2 distinct types of cells, the thyroid follicular cells (thyrocytes) and the parafollicular cells (calcitonin-producing cells or C cells). Thyrocytes secrete 2 hormones (triiodothyronine [T3] and thyroxine [T4]). The thyroid hormones act to affect the basal metabolic rate, protein synthesis, and synergy with growth hormone for growth and development in children. Secretion of T3 and T4 is regulated by thyroid-stimulating hormone (TSH), which is secreted from the anterior pituitary gland. TSH is regulated by thyrotropin-releasing hormone, which is produced by the hypothalamus. Parafollicular cells produce calcitonin in response to serum calcium homeostasis.^[1]^ In human embryos, an endothelial diverticulum in the midline of the ventral pharynx between the first and second pharyngeal pouches develops thyroid follicular cells, during the third or fourth week of gestation. Then, the thyroid bud starts its migration descending along the path of the thyroglossal duct from the tongue to the final location in front of the trachea at the seventh week of gestation.^[1,8]^ The C-cells are neuroendocrine cells in the thyroid. They are believed to be originated from the ultimobranchial bodies of the fourth pharyngeal pouch. The ultimobranchial bodies fuse with the primitive thyroid when thyroid migration is complete during the ninth week of gestation.^[8,9]^

The exact mechanism responsible for thyroid dysgenesis has not been completely known. The possible causes of thyroid dysgenesis include the impact of maternal antithyroid immunoglobulins, genetic mutations, and genes of transcription factors, such as *TITF1/NKX2–1*, *PAX8*, *FOXE1*, and *HHEX*.^[1,7]^ These transcription factors are not only expressed in functioning thyroid cells but also can be found in their precursors, which seem to be essential for the morphogenesis of early thyroid development. *TITF1/NKX2–1* is responsible for the thyroid-specific expression of thyroglobulin and thyroid peroxidase. *PAX8* plays a key role in the genetic regulatory cascade to control thyroid development. Mutation of *FOXE1* has been demonstrated the relation with thyroid migration in mice studies. However, no such mutation has been detected in the human ectopic thyroid up to now. Finally, *HHEX* is required to maintain the messenger RNA expression of the aforementioned transcription factor genes.^[3,8]^

The most common disorder of thyroid dysgenesis is ETT. ETT is the result of a failure of thyroid migration, not only along the path of the thyroglossal duct but also in the mediastinum or other distant subdiaphragmatic areas.^[10]^ Some cases of ETT function normally, but approximately one-third of patients have different levels of hypothyroidism. So surgical excision of ETT may lead to more severe hypothyroidism, requiring lifelong thyroid hormone replacement.^[5,11]^ ETT in hyperthyroidism is very rare, with only a few cases reported in the literature.^[3,12]^

The most common location of the ETT is at the base of the tongue, called a lingual thyroid, accounting for 90% of the reported cases.^[1–4]^ The first case of Lingual thyroid was described by Hickmann in 1869.^[13,14]^ More than 70% of the patients with lingual thyroid have no thyroid tissue in the normal location, as has seen in our case.^[5,15]^

Although 70% of patients with lingual thyroid have been reported with subclinical hypothyroidism, most cases of lingual thyroid are asymptomatic unless associated symptoms with severe hypothyroidism or progressive enlargement of gland size leading to several regional symptoms, such as dysphagia, foreign body sensation, or pain over the throat, dysphonia, hemoptysis, dyspnea, and upper airway obstruction.^[1,2]^ Enlargement of lingual thyroid tissue can be seen while experiencing upper respiratory tract infections associated with lymphoid tissue.^[16]^ Moreover, the size of lingual thyroid tissue may be enlarged by the stimulation of elevated TSH during puberty, pregnancy, or menstruation, when the demand for thyroid hormones increases that leads hypothyroidism to develop. This explains the reason why the reported lingual thyroid is more common in females.^[3,5,17,18]^ In addition, hypothyroidism and enlargement of a lingual thyroid may be induced by medications, such as lithium, which interferes with iodine metabolism and actions of TSH.^[19]^

Primary thyroid carcinomas arising in lingual thyroids are uncommon in less than 1% of cases.^[1,7]^ Usually, they are incidentally diagnosed after surgical excision of the ectopic mass. Reported pathological types are mostly papillary carcinomas, followed by follicular, mixed follicular and papillary, Hürthel cell, and medullary carcinomas.^[3,7,20]^

In diagnostic workup for lingual thyroid, thyroid function test should be performed. Thyroid function tests often reveal normal to low gland function with normal to decreased T4 and T3 levels, and elevated TSH levels.^[21]^ The most useful method to localize lingual thyroid is thyroid scintigraphy with ^123^I-iodine or ^99m^Tc-technetium, which shows the uptake of radionuclide activity at the tongue base and no activity in the normal location in the neck. Ultrasonography of the neck is a noninvasive and readily available tool in the evaluation of the presence or absence of thyroid tissue in orthotopic locations and conducting initial detection of the ETT. CT scan and MRI are also helpful to define the location and to evaluate the characteristics of ETT. FNAC can not only help to confirm the diagnosis of ETT but also to rule out the potential of malignancy.^[1,5,7]^

Although there is no consensus about the optimal therapeutic strategy for lingual thyroid, perhaps due to the clinical rarity, most authors agree the treatment depends on the severity of clinical symptoms and complications.^[1]^ Regular follow-up is required for completely asymptomatic patients with euthyroidism. While patients had hypothyroidism, exogenous thyroid hormone replacement is the mainstay of conservative treatment.^[1,21,22]^ Usually, it can not only correct hypothyroidism but also can effectively suppress and reduce the size of the enlarged lingual thyroid to improve the mild neck regional symptoms (airway obstruction, dysphagia, and dysphonia). Surgical excision is indicated when the neck regional symptoms could not be relieved by conservative treatment, especially in patients with severe, recurrent bleeding, significant airway obstruction, or malignancy.^[1,5]^ In order not to undergo lifelong hormone replacement therapy, the lingual thyroid can be excised and auto-transplanted to the muscles of the neck for patients with only the functional lingual thyroid and absent thyroid tissue in the neck.^[5,21,23,24]^ Thyroid ablation with radioactive iodine is an alternative to surgery for unfit patients or those who refuse to undergo surgical intervention.^[1,21]^

Our patient had no neck regional symptoms but presented with progressively hypothyroid symptoms. The amount of thyroid hormone secretion may not be enough for daily hormonal demand for her during these months until she sought medical advice. Once the patient was diagnosed with lingual thyroid with severe hypothyroidism through the clinical exam and ^99m^Tc thyroid scintigraphy, she started taking hormone replacement therapy, her symptoms were significantly improved during regular follow-up.

## 5 Conclusion

Lingual thyroid is a rare clinical entity that needs careful diagnostic workup including clinical examination, biochemical tests, imaging methods such as ultrasonography, scintigraphy, CT, MRI, and FNAC to plan the management. Lingual thyroid with hypothyroidism and no neck regional symptoms can be conservatively treated, however, regular follow-up is required for the prevention of potential risk of malignant transformation.

## Acknowledgments

The authors would like to thank all the people who participated in this study. The authors also are grateful to the hospital officials for providing support for this study.

## Author contributions

**Data curation:** Hsuan Huang.

**Investigation:** Hsuan Huang, Yi-Hsin Lin.

**Resources:** Yi-Hsin Lin.

**Writing – original draft:** Hsuan Huang.

**Writing – review & editing:** Yi-Hsin Lin.

## References

[1] Noussios G Anagnostis P Goulis DG Lappas D Natsis K. Ectopic thyroid tissue: anatomical, clinical, and surgical implications of a rare entity. Eur J Endocrinol 2011;165:375–82. 21715415 10.1530/EJE-11-0461

[2] Ibrahim NA Fadeyibi IO. Ectopic thyroid: etiology, pathology and management. Hormones (Athens) 2011;10:261–9. 22281882 10.14310/horm.2002.1317

[3] Santangelo G Pellino G De Falco N. Prevalence, diagnosis and management of ectopic thyroid glands. Int J Surg 2016;28:S1–6. 26708843 10.1016/j.ijsu.2015.12.043

[4] Sturniolo G Vermiglio F Moleti M. Thyroid cancer in lingual thyroid and thyroglossal duct cyst. Endocrinol Diabetes Nutr 2017;64:40–3. 27825535 10.1016/j.endonu.2016.07.010

[5] Thapa S Khanal P. Lingual thyroid with subclinical hypothyroidism in a young female. Case Rep Endocrinol 2021;2021:6693477. 33564479 10.1155/2021/6693477 PMC7867443

[6] Xu F Shao Z Yang G. The value of scintigraphy, computed tomography, magnetic resonance imaging, and single-photon emission computed tomography/computed tomography for the diagnosis of ectopic thyroid in the head and neck: a STROBE-compliant retrospective study. Medicine (Baltimore) 2018;97:e0239. 29595677 10.1097/MD.0000000000010239 PMC5895372

[7] Lukáš J Drábek J Lukáš D Zemanová I Rulseh A. Ectopic thyroid with benign and malignant findings: a case series. Int J Surg Case Rep 2020;66:33–8. 31790949 10.1016/j.ijscr.2019.11.011 PMC6909043

[8] De Felice M Di Lauro R. Thyroid development and its disorders: genetics and molecular mechanisms. Endocr Rev 2004;25:722–46. 15466939 10.1210/er.2003-0028

[9] Vandernoot I Sartelet H Abu-Khudir R Chanoine JP Deladoëy J. Evidence for calcitonin-producing cells in human lingual thyroids. J Clin Endocrinol Metab 2012;97:951–6. 22238389 10.1210/jc.2011-2772

[10] Basaria S Westra WH Cooper DS. Ectopic lingual thyroid masquerading as thyroid cancer metastases. J Clin Endocrinol Metab 2001;86:392–5. 11232030 10.1210/jcem.86.1.7130

[11] Ling L Zhou SH Wang SQ Wang LJ. Misdiagnosed ectopic thyroid carcinoma: report of two cases. Chin Med J (Engl) 2004;117:1588–9. 15498392

[12] Abdallah-Matta MP Dubarry PH Pessey JJ Caron P. Lingual thyroid and hyperthyroidism: a new case and review of the literature. J Endocrinol Invest 2002;25:264–7. 11936471 10.1007/BF03344002

[13] Hickman W. Congenital tumour of the base of the tongue passing down the epiglottis on the larynx and causing death by suffocation sixteen hours after death (sic.). Trans Path Soc Lond 1869;20:160.

[14] Kumar LK Kurien NM Jacob MM Menon PV Khalam SA. Lingual thyroid. Ann Maxillofac Surg 2015;5:104–7. 26389046 10.4103/2231-0746.161103 PMC4555932

[15] Kaushal D Shakrawal N Goyal A Nair NP Verma AK Prakash D. Ectopic lingual thyroid: an entity not to be missed!. Int J Mol Biol Open Access 2019;4:132–3.

[16] Kalnina M Pramalte A Zemniece L Safronov Y. Acute infectious thyroiditis in ectopic lingual thyroid causing dysphagia and dyspnoea: a case report and discussion. BJR Case Rep 2016;3:20160025. 30363312 10.1259/bjrcr.20160025 PMC6159276

[17] Thomas G Hoilat R Daniels JS Kalagie W. Ectopic lingual thyroid: a case report. Int J Oral Maxillofac Surg 2003;32:219–21. 12729787 10.1054/ijom.2002.0311

[18] Ramanathan R Veerapandian JP Sundari S. Lingual thyroid with hypothyroidism in a child. Int J Contemp Pediatr 2019;6:1747–9.

[19] Talwar N Mohan S Ravi B Andley M Kumar A. Lithium-induced enlargement of a lingual thyroid. Singapore Med J 2008;49:254–5. 18363010

[20] Massine RE Durning SJ Koroscil TM. Lingual thyroid carcinoma: a case report and review of the literature. Thyroid 2001;11:1191–6. 12186508 10.1089/10507250152741055

[21] Kumar SS Kumar DM Thirunavukuarasu R. Lingual thyroid-conservative management or surgery? A case report. Indian J Surg 2013;75:118–9. 24426535 10.1007/s12262-012-0518-4 PMC3693310

[22] Kansal P Sakati N Rifai A Woodhouse N. Lingual thyroid. Diagnosis and treatment. Arch Intern Med 1987;147:2046–8. 3675109

[23] Amr B Monib S. Lingual thyroid: a case report. Int J Surg Case Rep 2011;2:313–5. 22096763 10.1016/j.ijscr.2011.10.004 PMC3215257

[24] Koc G Taskaldiran I Aslan Felek S. Ectopic lingual thyroid presenting with massive hematemesis. Acta Endocrinol (Buchar) 2019;15:244–6. 31508184 10.4183/aeb.2019.244 PMC6711637

